# Performing IUI Simultaneously with hCG Administration Does Not Compromise Pregnancy Rate: A Retrospective Cohort Study

**Published:** 2018

**Authors:** Patsama Vichinsartvichai, Khanitta Traipak, Chirawattana Manolertthewan

**Affiliations:** - Infertility Unit, Department of Obstetrics and Gynecology, Vajira Hospital, Faculty of Medicine, Navamindradhiraj University, Bangkok, Thailand

**Keywords:** Artificial insemination, Postovulatory IUI, Pregnancy rate, Preovulatory IUI, Timing of hCG

## Abstract

**Background:**

The probability of conception occurs before ovulation in natural cycle, thus performing IUI before ovulation should not compromise the pregnancy outcomes.

**Methods:**

A retrospective cohort study was conducted at a university hospital during 2007 to 2015. The ovarian stimulation and monitoring were performed as usual. The total of 29 preovulatory IUI, and 221 postovulatory IUI couples were recruited. In postovulatory IUI, 5,000 *IU* of hCG was injected when dominant follicle reached 17 *mm*. The IUI was performed 36 to 40 *hr* afterward. In preovulatory IUI, hCG was injected and IUI was performed simultaneously when the dominant follicle reached the size. Data were compared using independent sample t test and Fisher’s exact test. A p-value of <0.05 was considered statistically significant.

**Results:**

The characteristics of both groups were comparable. The cumulative biochemical, clinical, and live birth rates were not different between prevulatory and postovulatory IUI groups (10.3% *vs*. 16.3%; p=0.407, 10.3% *vs*. 12.2%; p=0.77 and 10.3% *vs.* 11.3%; p=0.877, respectively).

**Conclusion:**

Performing IUI simultaneously with hCG administration does not compromise pregnancy rate.

## Introduction

Although success rate for intrauterine insemination (IUI) is relatively low (1) (around 10% per cycle), IUI is generally considered as the primary modality for infertility treatment. It involves mild ovarian stimulation or natural cycle and processed sperm insemination. The prognostic factors for success rate depend on women age (1), processed total motile sperm (1, 2), endometrium thickness (1), and number of dominant follicles (3).

Conventionally, human chorionic gonadotropin (hCG) is administered when dominant follicles are reached on ultrasonography. The IUI is then scheduled 36–40 *hr* afterward. However, the prob ability of conception occurs only during five days before and ends on the day of ovulation in natural cycle (4). Hence, the necessity of scheduled IUI after ovulation has been challenged. It is hypothesized that IUI can be scheduled on the same day as the dominant follicle achieves on ultrasonography without compromising the pregnancy outcome (5–10). The limitation of previous studies is the lack of powerful sample size to tell the difference among each treatment arm. The pooled data of the published studies might overcome this limitation (5–8, 10–13).

The aims of the present study were to compare the pregnancy outcomes between preovulatory and postovulatory IUI to study the pregnancy outcomes between such timing of hCG and IUI.

## Methods

### Study design and participants:

The retrospective cohort study was carried out at the infertility clinic in a tertiary-care university hospital. The study was conducted in accordance to the ethical principles of the Declaration of Helsinki, and the Vajira Institutional Review Board approved the study protocol.

All data were collected from IUI cycles between October 2007 to October 2015, after excluding the couples that were diagnosed with severe male factor infertility and bilateral tubal obstruction. Totally, 29 preovulatory IUI, and 221 postovulatory IUI couples were recruited. The patients were diagnosed with endometriosis, or endometrial polyp by laparoscopic or hysteroscopic surgery with histopathologic confirmation.

### Ovarian stimulation:

In each IUI cycle, ovarian stimulation was performed either by clomiphene citrate (CC) alone (Ovinum, Biolab, Samutrprakarn, Thailand), gonadotrophin alone (Menopur, Ferring, Saint-Prex, Switzerland), or combination of CC and gonadotrophin, based on physician’s discretion starting on the 3rd to 5th day of menstrual cycle. The growth of follicles and endometrial response were monitored by transvaginal ultrasonography on cycle days 9 to 14.

### Timing of hCG injection:

The standard protocol in our unit had been to inject 5,000 *IU* of hCG (Pregnyl, MSD, New Jersey, USA) when at least one dominant follicle reached 17 *mm* in mean diameter. The IUI was performed 36 to 40 *hr* after hCG administration. During October 2007 to December 2014, all patients were treated according to the standard protocol (postovulatory group). From January 2015, hCG was administered and IUI was performed on the same day when at least one dominant follicle reached 17 *mm* in mean diameter (preovulatory group).

### Sperm preparation and IUI procedure:

The semen samples were collected by masturbation following an abstinence of 3–5 days and then left to be liquefied in the 37°*C* incubator for 30 *min*. A droplet of undiluted semen was analyzed according to 5th edition of WHO laboratory manual (14). After semen analysis, the samples were centrifuged at 300 *g* for 15 *min* in a 45% and 90% Sil-Select-STOCK sperm preparation medium (FertiPro, Beernem, Belgium). The supernatant was discarded and the pellet was washed twice in sperm washing medium (FertiCult Beernem, Belgium) at 250 *g* in 5 *min* in each washing. The prepared sperm was kept in the 37°*C* incubator until used.

The prepared sperm was inseminated into the uterus through the cervix using an in-house compile soft IUI catheter.

### Patient follow:

Two weeks after the IUI procedure, the urine pregnancy test (UPT) was done. In case of positive UPT, a serum level of β-hCG is often determined for the confirmation of biochemical pregnancy. For the clinical pregnancy, the vaginal sonography was performed every 2 weeks after the positive pregnancy result to inspect the gestational sac and fetal heart beat activity until gestational age of 12 weeks.

### Statistical analysis:

All data were analyzed by SPSS software (version 22.0). Data were presented as mean±standard deviation (SD), number (%), or percentage (95% confidence interval, CI), as appropriate. Data comparisons were analyzed using independent sample t test for continuous data and *χ*^2^ or Fisher’s exact test as appropriate for categorical data. The binary logistic regression analysis adjusted for duration of infertility, number of dominant follicles, endometrium thickness, and total motile sperm count were used to compare the pregnancy rates among preovulatory and postovulatory IUI group. A p-value of <0.05 was considered statistically significant.

## Results

Between October 2007 and December 2014, there were 466 IUI cycles from 221 couples who underwent IUI after 36–40 *hr* of hCG injection (postovulatory group) while between January 2015 and October 2015, there were 54 IUI cycles from 29 couples who underwent IUI on the same day of hCG injection (preovulatory group). The baseline characteristics of both groups were comparable (Table 1). The average women age was 35 years, and mean duration of infertility was 3.5±2.5 years in preovulatory group and 3.8±2.7 years in postovulatory group, respectively. The major etiologies of infertility were unexplained, endometriosis, and anovulation, respectively. No mild male factor infertility was observed. Most of the cycles were stimulated with clomiphene citrate alone. However, mean total motile sperm count after sperm preparation in postovulatory IUI group was significantly higher than preovulatory IUI group (56×10^6^/*ml* versus 23×10^6^/*ml*, respectively, p<0.001).

**Table 1.** Baseline characteristics of preovulatory and postovulatory IUI groups. Data are presented as mean± standard deviation or number (%)

	**Preovulatory IUI (n=29)**	**Postovulatory IUI (n=221)**	**p**
**Age (years)**	34.7±4.8	35.0±4.4	0.758^*^
**Duration of infertility (years)**	3.5±2.5	3.8±2.7	0.542^*^
**Causes of infertility**			<0.001^**^
Endometriosis	5 (17.2)	60 (27.1)	
Endometrial polyp	4 (13.8)	2 (0.9)	
Anovulation	4 (13.8)	21 (9.5)	
Unexplained infertility	8 (27.6)	125 (56.6)	
Number of cycle	1.9±0.8	2.2±1.6	0.291^*^
**Stimulation cycle characteristics**			
**Stimulation protocols**			0.341^**^
CC alone	27 (93.1)	194 (87.8)	
CC + FSH	0 (0)	15 (6.8)	
FSH alone	2 (6.9)	12 (5.4)	
**Number of dominant follicle**	1.7±0.8	1.4±0.7	0.03^*^
**Size of largest dominant follicle (*mm*)**	21.1±3.4	20.4±3.2	0.256^*^
**Endometrium thickness (*mm*)**	8.4±2.0	8.0±2.4	0.43^*^
**Processed total motile sperm count (×10^6^/*ml*)**	23.1±18.2	56.1±48.6	<0.001^*^

* Data were analyzed using independent sample t-test;

** Data were analyzed using the *χ*^2^ test; IUI: intrauterine insemination; CC: Clomiphene citrate; FSH: Follicle stimulating hormone

### Pregnancy outcomes:

The pregnancy outcomes were displayed in table 2. The biochemical pregnancy rates were comparable among both groups (10.3%, 95% CI 1.0–22.0, and 16.3%, 95% CI 11.0–21.0, p=0.587 in preovulatory IUI, and postovulatory IUI group, respectively). These biochemical pregnancies resulted in 3 live-births in preovulatory IUI group (10.3%, 95% CI 1.0–22.0), and 25 live-births in postovulatory IUI group (11.3%, 95% CI 7.0–16.0). The live-birth rates among both group were not different (p=1.000). There was no miscarriage in preovulatory IUI group while there were 11 miscarriages in postovulatory IUI group (5.0%, 95% CI 2.0–8.0, p= 0.622). The binary logistic regression was applied to adjust for the effect of duration of infertility, number of dominant follicles, endometrium thickness, and total motile sperm count to the pregnancy outcomes among both IUI group as represented in table 3. There was no significant effect of timing of IUI to the pregnancy results.

**Table 2.** Comparison of pregnancy rate per couple with different timing in hCG injection. Data are presented as number (%)

	**Preovulatory IUI (n=29)**	**Postovulatory IUI (n=221)**	p^*^
**Biochemical pregnancy rate**	3 (10.3)	36 (16.3)	0.587
**Clinical pregnancy rate**	3 (10.3)	27 (12.2)	1.000
**Live birth rate**	3 (10.3)	25 (11.3)	1.000
**Miscarriage rate**	0 (0)	11 (5.0)	0.622

* Data were analyzed using the Fisher’s exact test; IUI: intrauterine insemination

**Table 3.** Binary logistic regression analysis of pregnancy rate per couple among preovulatory and postovulatory IUI group (Postovulatory IUI is the reference group)

	Adjusted OR (95% CI)^*^	**p**
**Biochemical pregnancy rate**	0.539 (0.144–2.024)	0.360
**Clinical pregnancy rate**	0.804 (0.208–3.107)	0.752
**Live birth rate**	0.796 (0.205–3.096)	0.742

* Adjusted for duration of infertility, number of dominant follicles, endometrium thickness, and total motile sperm count

## Discussion

The concept of performing IUI after expected ovulation (36–40 *hr* after hCG administration) has been challenged for decades. Performing IUI before expected time of ovulation (preovulatory IUI) should not decrease the pregnancy rate or even increase the chance of pregnancy according to many reasons. Firstly, the ovulation occurs 24–56 *hr* after LH surges (median 36 *hr*) in natural cycles (15). On the other hand, the ovulation prevails 36–48 *hr* after hCG administration (16, 17). However, the premature LH surges occur in stimulated IUI cycle (18), which contributes to spontaneous ovulation about 24 *hr* after the surges (19). The conventional postovulatory IUI might be too late if premature LH surges occurred while preovulatory IUI still makes fertilization possible. Secondly, the cycle fecundability in natural cycle is maximum when the intercourse takes place between two days before ovulation, and the day of ovulation (4).

In our data set, biochemical pregnancy rate and clinical pregnancy rate in preovulatory IUI were lower than those in postovulatory IUI but not significantly different (10.3% versus 16.3%, p=0.407, and 10.3% versus 12.2%, p=0.77, respectively). The live-birth rate was not different among both groups (10.3% versus 11.3%, p=0.877, in preovulatory, and postovulatory IUI, respectively) according to higher miscarriage rate in postovulatory IUI (0.0% versus 5.0%, p=0.219, in preovulatory, and postovulatory IUI, respectively).

The characteristics of relevant publications were summarized in table 4. Fuh et al.’s investigated the time-related manner between spontaneous endogenous luteinizing hormone surge, hCG administration, and pregnancy rates in IUI cycles (20). Propst et al. (2012) used the same data set as Propst et al. (2007) did which might consequently deviate from the data in the previous studies.

**Table 4.** Overview and characteristics of studies discussing IUI and timing of hCG administration

**Author, year**	**Location**	**Study type**	**Patients**	**Intervention**
**Fuh et al., 1997**	Australia	Retrospective cohort	N=463, 1990–1995	Group A, endogenous LH surge; group B, hCG after LH surge; group C, hCG before LH surge
**Robb et al., 2004**	USA	Retrospective cohort	N=90, 2000–2001	IUI performed 24 *hr* versus 36 *hr* after hCG injection
**Wang et al., 2006**	Taiwan	NA	N=135	Group 1, IUI 24 *hr* after hCG; group 2, IUI 36 *hr* after hCG
**Propst et al., 2007**	USA	RCT	N=206,	IUI performed 12 *hr* versus 36 *hr* after hCG injection
**Järvelä et al., 2010**	Finland	Retrospective cohort	N=233, 2007–2009	IUI performed 24 *hr* versus 36 *hr* after hCG injection
**Rahman et al., 2011**	India	RCT	N=204	IUI performed 24 *hr* versus 36 *hr* after hCG injection
**Propst et al., 2012**	USA	RCT	N=213	IUI performed 12 *hr* versus 36 *hr* after hCG injection
**Aydin et al., 2013**	Turkey	RCT	N=220, 2011–2013	IUI simultaneously versus 34–36 *hr* after hCG injection
**Dehghani-Firouzabai et al., 2014**	Iran	RCT	N=100	IUI simultaneously versus 34–36 *hr* after hCG injection
**Mostafa et al., 2014**	Egypt	RCT	N=100, 2010–2011	IUI simultaneously versus 24–32 *hr* after hCG injection

RCT: Randomized controlled trial; N: Number of couples; NA: Not available

From our study, unexplained infertility was the major cause of infertility in postovulatory IUI group while it was only about one fourth in preovulatory IUI (56.6% versus 27.6%, respectively). From previous studies, they found that spontaneous pregnancy rates in these patients were high up to 15% in one year, and might be up to 35% in two years (22). These might explain the reason for better biochemical and clinical pregnancy rates in our postovulatory IUI group. However, the duration of infertility in our patients was higher than 3 years in both groups, which was a poor prognostic predictor for fecundability (23). Furthermore, invasive investigations such as diagnostic hysteroscopy and diagnostic laparoscopy were not performed in every patient. The combined diagnostic surgeries can reveal the concealed pelvic pathology in 50% of unexplained infertility patients (24).

The pregnancy rate in IUI treatment improves with an increased processed total motile sperm count (1, 2, 25). It can predict clinical pregnancy rate better than sperm chromatin dispersion test (26) with the threshold concentration of 10×10^6^/*ml* (27). Processed total motile sperm count in our study is higher than the threshold in both groups but significantly higher in postovulatory IUI (23.1 ×10^6^ versus 56.1×10^6^/*ml*, p<0.001 in preovulatory IUI, and postovulatory IUI, respectively). These also explain the higher biochemical, and clinical pregnancy rate in postovulatory IUI but not live-birth rate.

The higher miscarriage rate in postovulatory IUI than preovulatory IUI in our data can be explained by our hypothesis that premature LH surges occurred more frequently in postovulatory IUI group. These findings are also in line with previous studies that higher LH concentration at the time of maximum follicular growth was associated with lower conception, and may contribute to higher early pregnancy loss in PCOS patients (28). Unfortunately, the direct correlation of premature LH surges and adverse pregnancy outcomes has not been explored. It was postulated that premature LH surges may give rise to the sequence of events that leads to pregnancy loss. First, it stimulates premature cumulus expansion, premature initiation of oocyte meiosis, and postmature oocyte at the time of fertilization (21). The postmature oocytes are found to have lower rate of normal fertilization, lower cleavage rate, and more than 1 pronuclear or pronuclear asynchrony (29). Second, premature progesterone secretion that appears after premature ovulation results in advanced endometrial dating, endometrium-embryo asynchrony and change in implantation window (21). Thus, more abnormal embryos and poor endometrium receptivity affect the pregnancy loss.

Our study has some limitations. Firstly, the retrospective nature of the study limits the variables that could be analyzed such as hormone monitoring, and the unmatched sample size among both groups. Secondly, the heterogeneity among each included study existed such as the timing of IUI after hCG administration in preovulatory IUI group which varied from simultaneously with IUI, 12 *hr*, and 24 *hr*. The outcome measures also varied among each study. Thus, these discrepancies made it difficult to perform a meta-analysis.

## Conclusion

It seems preovulatory IUI gives comparable pregnancy outcomes as postovulatory IUI. Performing IUI on the same day as hCG injection has numerous benefits. For patients, the lower number of clinic visits, and flexible time of hCG injection contribute to less stress, and more flexible treatment cycle. For the clinic, this protocol provides a flexible schedule for sperm preparation and IUI. However, a larger study with enough power to detect the live-birth rate difference with our protocol should be performed.
